# Design of an observational multi-country cohort study to assess immunogenicity of multiple vaccine platforms (InVITE)

**DOI:** 10.1371/journal.pone.0273914

**Published:** 2022-09-15

**Authors:** Irini Sereti, Kathryn Shaw-Saliba, Lori E. Dodd, Robin L. Dewar, Sylvain Laverdure, Shawn Brown, Olivier Tshiani Mbaya, Jean Jacques Muyembe Tamfum, Placide Mblala-Kingebeni, Ydrissa Sow, Esther Akpa, Mory Cherif Haidara, Karine Fouth Tchos, Abdoul Habib Beavogui, Aaron Neal, Dona Arlinda, Dewi Lokida, Louis Grue, Mary Smolskis, Laura A. McNay, Dehkontee Gayedyu-Dennis, Guillermo M. Ruiz-Palacios, Abelardo Montenegro-Liendo, Moctar Tounkara, Seydou Samake, Ganbolor Jargalsaikhan, Delgersaikhan Zulkhuu, Shera Weyers, Tyler Bonnett, Gail E. Potter, Randy Stevens, Adam Rupert, Jamila Aboulhab, Jean-Luc Biampata, Alexandre Delamo, Bienvenu Salim Camara, Herman Kosasih Indonesia, Muhammad Karyana, James T. Duworko, Justino Regalado-Pineda, Paola del Carmen Guerra-de-Blas, Seydou Doumbia, Djeneba Dabitao, Naranjargal Dashdorj, Naranbaatar Dashdorj, Kevin Newell, Alyson Francis, Kevin Rubenstein, Victoria Bera, Iman Gulati, Ratna Sardana, Monica Millard, Renee Ridzon, Sally Hunsberger

**Affiliations:** 1 Division of Intramural Research, National Institute of Allergy and Infectious Diseases, National Institutes of Health, Bethesda, MD, United States of America; 2 Division of Clinical Research, National Institute of Allergy and Infectious Diseases, National Institutes of Health, Bethesda, MD, United States of America; 3 Virus Isolation and Serology Laboratory, Frederick National Laboratory for Cancer Research, Frederick, Maryland, United States of America; 4 Laboratory of Human Retrovirology and Immunoinformatics, Frederick National Laboratory for Cancer Research, Frederick, Maryland, United States of America; 5 AIDS Monitoring Laboratory, Frederick National Laboratory for Cancer Research, Frederick, Maryland, United States of America; 6 Clinical Monitoring Research Program Directorate, Frederick National Laboratory for Cancer Research, Frederick, Maryland, United States of America; 7 Institut National de Recherche Biomedicale, Kinshasa, Democratic Republic of Congo; 8 Centre National de Formation et de Recherche en Santé Rurale de Maferinyah, Maférinya, Guinea; 9 Ministry of Health, Jakarta, Indonesia; 10 Indonesia Research Partnership on Infectious Diseases National Institute of Health Research and Development, Jakarta, Indonesia; 11 Partnership for Research on Vaccines and Infectious Diseases in Liberia, Monrovia, Liberia; 12 Departamento de Infectología, Instituto Nacional de Ciencias Médicas y Nutrición Salvador Zubirán, Ciudad de México, Mexico; 13 The Mexican Emerging Infectious Diseases Clinical Research Network, Ciudad de México, Mexico; 14 University Clinical Research Center, University of Sciences, Techniques and Technologies of Bamako, Bamako, Mali; 15 The Liver Center, Mongolia and Onom Foundation, Ulaanbaatar, Mongolia; 16 Subdirección de Medicina, Instituto Nacional de Enfermedades Respiratorias Ismael Cosío Villegas, Ciudad de México, Mexico; 17 Systex, Inc, Rockville, Maryland, United States of America; Public Library of Science, UNITED KINGDOM

## Abstract

In response to the COVID-19 pandemic, COVID-19 vaccines have been developed, and the World Health Oraganization (WHO) has granted emergency use listing to multiple vaccines. Studies of vaccine immunogenicity data from implementing COVID-19 vaccines by national immunization programs in single studies spanning multiple countries and continents are limited but critically needed to answer public health questions on vaccines, such as comparing immune responses to different vaccines and among different populations.

## Introduction

The COVID-19 global pandemic was declared on March 11, 2020, and as of April 30, 2022, there have been over 500 million confirmed cases of COVID-19, including more than six million deaths, reported to the World Health Organization (WHO). The development of multiple COVID-19 vaccines was a major milestone in the course of the pandemic [1]. Currently, the WHO has granted emergency use listing (EUL) to 11 vaccines: 4 non-replicating viral vector vaccines (Johnson & Johnson/Janssen, Ad26.COV2.S/Oxford/AstraZeneca/Vaxzevria, Serum Institute of India Covishield [AstraZeneca formulation] and CanSino, Convidecia); 3 inactivated virus vaccines (Sinopharm/Covilo, Bharat Biotech Covaxin, and Sinovac/CoronaVac); 2 mRNA vaccines (Moderna/Spikevax and Pfizer/BioNTech/Comirnaty); and 2 protein subunit vaccines (Novavax/Nuvaxovid and Serum Institute of India COVOVAX [Novavax formulation]) [2]. As of April 27, 2022, 11.4 billion vaccine doses have been administered globally. Global vaccination coverage (people fully vaccinated with last dose of a primary series) lies at 43% but varies widely, with up to 73% coverage in high- and upper middle-income regions to 50% in lower middle-income and 13% in low-income areas [3]. In many countries, booster doses of vaccines (with additional one or two doses after the initial regimen) have also been rolled out in addition to initial vaccine regimens, with 12% of the population boosted globally.

Most observational COVID-19 vaccine studies conducted to-date have been limited in scope to individual countries, relying on data solely from vaccine platforms available in-country [3,4]. Countries from the African Region, particularly western and central Africa, remain underrepresented [1]. Immunogenicity data from single studies spanning multiple countries and continents, further strengthened by standardized and centralized testing at a single laboratory, are limited but critically needed.

## Materials and methods

### Ethical statement

Each country serves as a separate local sponsor of the study and its related country-specific activities. The main protocol and each of the site-specific appendices received approval from local ethics committees and/or health authorities prior to study implementation including Democratic Republic of the Congo (DRC)—School of Public Health of Kinshasa Ethical Committee—ESP/CEA43C/2021–27 July 2021, Indonesia—Tangerang District Hospital Health Research Ethics Committee—# 445/011- KEP-RSUTNG– 30 July 2021, Liberia—The National Research Ethics Board (NREB)–Ref: NREB-006-21 –August 5, 2021, Mali—University of Science, Bamako Techniques and Technology–#2021/213/USTTB—Sept 2, 2021, Guinea—CNERS—#: 137/CNERS/21 –Sept 8, 2021, Mongolia—Ministry of Health Independent Ethics committee—# 265–25 Jan 2022, and Mexico—COFEPRIS - 213300410A0205/2022 –March 24, 2022. All study participants must sign an informed consent in a language they understand prior to initiate of any study procedures.

### Clinical study design

#### Study rationale

To meet this need, the International Study in COVID Vaccines to Assess Immunogenicity, Reactogenicity, and Efficacy (InVITE) study was developed and initiated in August of 2021 (https://clinicaltrials.gov/ct2/show/NCT05096091). This large observational cohort study includes 7 countries, the Democratic Republic of the Congo (DRC), Guinea, Indonesia, Liberia, Mali, Mexico, and Mongolia. These countries were chosen based on existing infrastructure and ongoing or newly established collaborations with the National Institute of Allergy and Infectious Diseases in the US. The aim of the study is to evaluate immunogenicity of COVID-19 vaccines administered as part of each country’s national COVID-19 immunization program (Table 1).

**Table 1:** Participating countries and vaccines that are available in each participating country.

	DRC	Guinea	Indonesia	Liberia	Mali	Mexico	Mongolia
**mRNA**							
Moderna							
Pfizer							
**Inactivated** **Virus**							
Sinovac							
Sinopharm							
**Adenovirus**							
Astrazeneca							
CanSino							
Covishield							
J&J							
SK Bio							
Sputnik V							
**Protein** **Subunit**							
Novavax							

The InVITE study is continuing to collect data from up to 6,000 participants for up to 2 years of follow-up to evaluate immunogenicity, durability of immunity, and breakthrough infections in persons vaccinated, including data relevant to each country as well as the international community.

### Study objectives

The primary objective of the study is to characterize immunogenicity of available COVID-19 vaccines at 2 months after completion of the initial or booster vaccine regimen, separately for each regimen and country. Secondary objectives are to evaluate the durability of immunogenicity, assess immunogenicity in predefined subgroups (age, sex, BMI (body mass index), comorbidities, HIV infection, pregnancy, malaria or active tuberculosis (TB), or evidence of prior infection with SARS-CoV-2), sequence SARS-CoV-2 viruses detected during breakthrough infections, compare immunogenicity between different vaccine regimens and countries, evaluate sero-incidence of infection, and evaluate symptomatic infection rates after vaccination to the extent possible based on symptomatic visits. At the time of protocol writing, an immunologic threshold or correlate of protection has not been established. If a correlate of protection is not established at the time of analysis, a cut point to distinguish high versus low levels of antibodies will be defined in the statistical analysis plan (SAP) prior to analysis, based on scientific literature and/or data outside of this study. Combined, cross country data for each vaccine will also be examined.

### Eligibility

Adults ≥18 years of age who are receiving either their initial or booster COVID-19 vaccine regimen according to country vaccination guidelines are eligible to enroll. Initial vaccine recipients and booster recipients are enrolled as separate cohorts. Initial vaccine recipients must be vaccine-naïve and are enrolled within one day of receipt of their first COVID-19 vaccine. Booster recipients must have documented completion of a prior vaccine regimen and are enrolled within one day of booster vaccine receipt. Enrollment occurs following written informed consent. Candidates are excluded if they are participating in a COVID-19 vaccine clinical trial or are unable to attend follow-up study visits or procedures. During the baseline enrollment visit, age, sex, BMI, targeted physical exam, comorbidities (diabetes, hypertension), HIV infection, pregnancy, malaria, TB, evidence of prior infection with SARS-CoV-2 (based on anti-nucleocapsid antibody detection in those who have not previously received whole inactivated virus vaccines) and previous COVID-19 vaccinations (for participants receiving boosters) are recorded. Optional HIV testing is offered to all participants, and optional pregnancy testing is offered to women of childbearing age. Pregnant women are excluded from participation in countries where local policies restrict COVID-19 vaccination for pregnant women. Study visits occur at months 2-, 10-, 16-, and 22-months post-vaccine regimen within defined windows. Visits that occur out of the window periods are permitted, within reasonable timeframes, to avoid missed visits. Serum, whole blood, and dried blood spots are collected for the immunological analyses at all study visits.

Subgroup analyses will be based on age, sex, BMI, comorbidities, HIV infection, pregnancy, malaria, or TB and prior infection with SARS-CoV-2 with comparisons within each vaccine regimen. The impact of malaria, dengue, and helminth infections on immune response will be evaluated serologically given that recent data indicate that cross-reactivity has been observed on SARS-CoV-2 serological assays [5–9]. Malaria will be specifically tested for, as there is evidence that indicates it can suppress the immune response to some vaccines [5]. To help understand cross-reactivity, pre-pandemic specimens will also be evaluated to assess any background responses on the serological assays.

### Study primary and secondary end points

The study’s primary endpoint is the immune response measured by the level of anti-Spike IgG antibody (anti-S Ab) at 2 months after the completion of the initial vaccine regimen or booster vaccine regimen. The secondary endpoints include anti-S Ab measured at 10-, 16-, and 22-months post-vaccine regimen to evaluate duration of immunogenicity, anti-S Ab in predefined subgroups. Additionally, incidence of symptomatic infection will be tracked among participants who present for sick visits and test positive for acute SARS-CoV-2 infection.

The primary immunogenicity marker, anti-S Ab will be measured by a sensitive quantitative assay. Sero-incidence of infection will be estimated with measurement of anti-nucleocapsid antibody at each timepoint as a marker of prior SARS-CoV-2 infection for persons whose vaccines did not contain inactivated virus. For infections detected at the symptomatic visits, full genome SARS-CoV-2 viral sequencing will be performed, and live virus or surrogate neutralization assays may be considered to assess neutralization titers and impact of novel variants on neutralizing titers. These neutralization assays have been used to evaluate responses in acute infection studies, therapeutic trials and vaccine studies [10–13]. Serological assays will be checked for background cross-reactivity using pre-pandemic specimens from countries endemic for malaria. If cross-reactivity is observed, methods to correct for the cross-reactivity will be utilized [14].

### Site-specific appendices

Although all countries are following the same main study protocol and visit schedule, countries have the flexibility to add objectives, blood draws, and even study visits as shown in Table 2. For example, in Indonesia, additional study visits and blood draws have been included between main study visits to examine antibody titers at shorter intervals after vaccine administration. These changes have been outlined in a site-specific appendix to the main protocol. In Mongolia, given a significant prevalence of viral hepatitis and chronic liver disease, baseline data include screening for chronic viral hepatitis, assessment of liver function, and liver fibrosis. These data will be used to assess the impact of underlying chronic liver diseases including cirrhosis on immune responses to vaccines.

**Table 2:** Country-specific information related to enrollment and follow-up and planned local studies.

Country	Target Enrollment	Vaccines Included	Vaccine Regimen	Study sites	Recruitment Methods	Participant Follow-up	Additional Local Studies
DRC	1,100	• Moderna • Pfizer • J&J • Sinovac	• Initial	• Saint Joseph Referral Hospital, Kinshasa	• Outreach at the hospital COVID-19 vaccination site	• Weekly telephone calls • Home visits, as permitted and needed	• None
Indonesia	700	• Moderna • Pfizer • AstraZeneca • Sinovac • Novavax	• Initial • Booster	• Tangerang Public Hospital, Tangerang • TC Hillers Public Hospital, Maumere, • Dr. H Moch Ansari Saleh Regional Hospital, Banjarmasin	• HCWs and hospital staff approached and invited to the study. • Recruitment at primary health care and vaccination centers	• Telephone calls every two weeks • Home visits, as permitted and needed	• Additional visits for additional SARS-CoV-2 serology testing at the time of 2^nd^ vaccination, 4–5 months after vaccination, and 6–7 months after vaccination
Guinea	500	• Moderna • Pfizer • AstraZeneca • J&J • Sinovac • Sinopharm	• Initial	• Maferinyah Research Health Center	• Outreach at vaccination site • Mobile community outreach	• Telephone calls prior to study visits • Home visits, as permitted and needed	• None
Liberia	700	• Pfizer • J&J	• Initial • Booster	• PREVAIL Duport Road Research Site, Monrovia	• Focal Group discussions during mobile vaccination efforts • Outreach at vaccination sites	• Telephone calls several times during the week before study visits • Home visits, as permitted and needed	• None
Mali	800	• Pfizer • AstraZeneca • J&J • Sinopharm • Sinovac	• Initial	• Yirimadio Primary Health Care Center, Bamako • Taliko Primary Health Care Center, Bamako • University Clinical Research Center, University of Sciences, Techniques and Technologies of Bamako	• Outreach at vaccination centers at each of the study sites	• Weekly telephone calls	• None
Mexico	600	• Moderna • Pfizer • AstraZeneca • Sputnik V	• Initial • Booster	• National Institute of Medical Sciences and Nutrition “Salvador Zubirán”, Mexico City • National Institute of Respiratory Diseases “Ismael Cosío Villegas”, Mexico City • Hospital “Dr. Manuel Gea González”, Mexico City • General Hospital "Dr. Aurelio Valdivieso”, Oaxaca • High Speciality Regional Hospital “Ciudad Salud”, Tapachula, Chiapas	• Outreach at vaccination centers	• Telephone calls, email, or text message ranging from once per week to once per month, depending on participant choice	• None
Mongolia	1000	• Pfizer • AstraZeneca • Sinopharm	• Booster	• Liver Center Clinic, Ulaanbaatar	• Social media • Zoom calls	• Weekly text messages • Text message reminders for upcoming visits	• Impact of chronic viral hepatitis on immune response to COVID-19 vaccine

### Study visits

**Fig 1:**
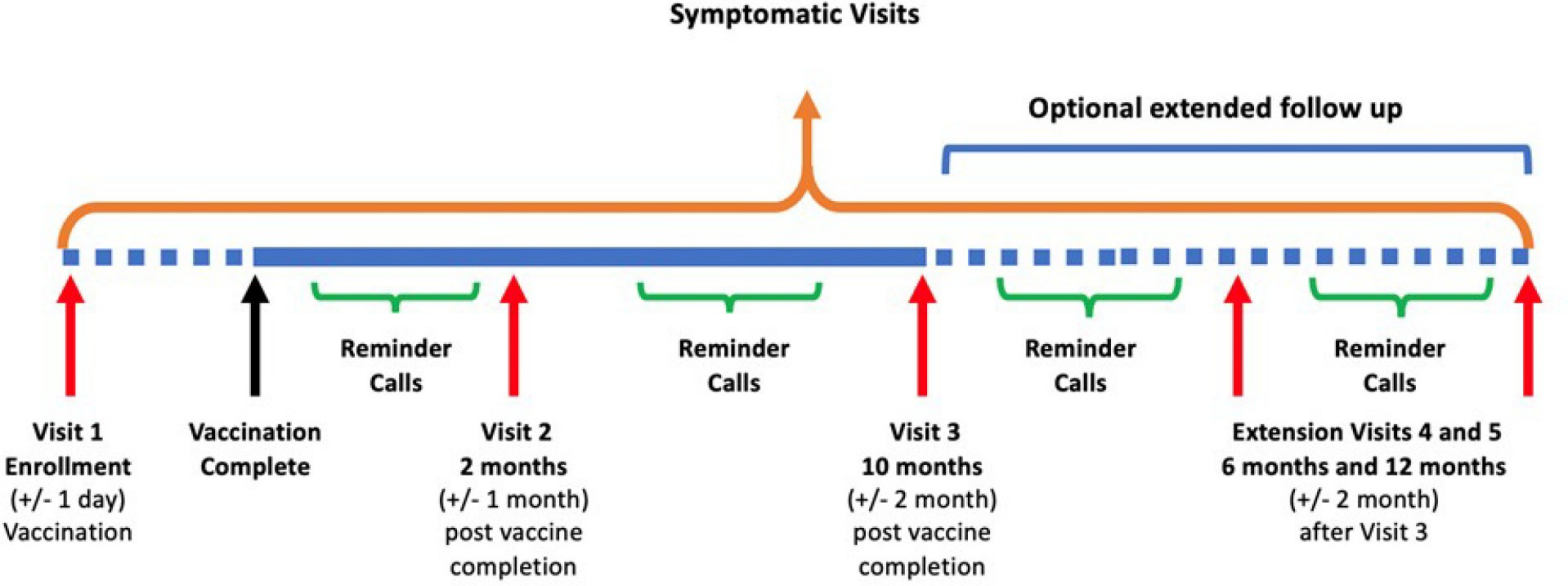
Study schematic.

A schematic of the study visit timing is shown in Fig 1. The last two study visits are optional and included in Version 2.0 of the protocol and informed consent. At each study visit, participants have vital signs checked, have a blood sample collected and their medication list updated, are asked if they had COVID-19 since the last visit, and if they received any additional vaccine doses.

As part of the initial visit, participants are instructed on the symptoms consistent with COVID-19 and are asked to return to the study site for evaluation at any point of time following enrolment if they develop any ot these. During these “symptomatic visits,” nasal or oral swabs are collected for SARS-CoV-2 detection according to local standard-of-care practices, a mid-turbinate swab is collected for research testing and SARS-CoV-2 sequencing, and blood is drawn for subsequent study-specific immunogenicity testing. Study staff communicate regularly, weekly or twice monthly, if possible, with study participants via phone calls or text messaging to remind them of scheduled visits and the importance of reporting symptoms consistent with SARS-CoV-2 infection (Table 2).

### Statistical aspects

The primary analysis will estimate the proportion of participants who achieve an immunologic response to vaccine at 2 months (Visit 2) after completion of the vaccine regimen separately for each country and vaccine regimen. For the initial vaccine regimens, there will be separate analyses based on enrollment (Visit 1) serology group (positive or negative SARS-CoV-2 serology). For the booster regimens, the analyses will be grouped based on Visit 1 antibody levels specified in the statistical analysis plan (SAP). The primary analysis will include only those who completed their vaccination regimen and had blood samples collected at both Visit 1 and Visit 2. It is assumed that at baseline, the largest baseline serology group contains 70% of the participants. The sample size is based on examining the half-width of the estimated 95% confidence intervals (CIs), assuming an immunologic response rate of 50%. Table 3 displays sample sizes to account for the range of numbers of participants receiving each vaccine regimen by country. For context, see Table 2, which displays vaccine regimens included in this study, by country. For example, in Mali, over 400 people receiving J&J as their initial vaccine have enrolled, so if 70% of them complete vaccinations, are seronegative at baseline, and contribute Visit 2 antibody data, then the half-width of the CI for the proportion of seronegative people with immunological response is expected to be around 6.0%. In Guinea, however, around 100 received this vaccine, so we expect a CI half-width around 12.1%.

**Table 3:** Half widths of 95% CI for estimates of the immunologic response rate using various sample sizes.

Half-width (%)	Sample size
17.0	50
12.1	100
8.5	200
6.9	300
6.0	400
5.4	500
4.4	750
3.8	1000
3.1	1500

A detailed Statistical Analysis Plan (SAP) has been prepared for the overall study (see supporting information) that provides guidance on general concepts on how the data will be analyzed. Additional manuscript-specific SAPs will be prepared prior to analysis of the data and submitted as supplements along with each manuscript submission. In general, statistical methods will include 95% CIs using the Clopper Pearson method and logistic regression for binary data. T-tests and mixed effects models with log transformation of the antibody response values will be used for continuous antibody level data.

### Study conduct, data management and operational logistics

The InVITE study leadership consists of a core team of physicians, statisticians, and a laboratory research scientist from the Division of Clinical Research (DCR) of the National Institute of Allergy and Infectious Diseases (NIAID). Country-specific study activities are overseen by a DCR team of special project (SP) leads for all countries except Mongolia, who works directly with InVITE study leadership. The number of InVITE study sites within each country range from one to seven, and all sites are equipped with clinical research staff and infrastructure supported by DCR (Table 2). Site selection was based on previous experience, vaccine national program rollout logistics and prevalence of comorbidities of interest such as malaria. For each country, the Principal Investigators (PIs) were selected based on their clinical research experience, availability, and interest in being accountable for the study activities at their respective sites.

Throughout the study, regular teleconferences are held and include InVITE study leadership, country InVITE scientific/medical teams, laboratory, and data management teams. In-country study teams play crucial roles to provide guidance and assist with regulatory and ethics protocol approvals, including material transfer agreements, import permits for supplies, staffing, training, and laboratory needs. Monthly investigator meetings are held as a forum for information exchange on topics such as recruitment and visit adherence as well as providing feedback and suggestions on study procedures. In addition, an InVITE Publications Policy has been developed to guide the drafting and review of all study-related abstracts, presentations, and manuscripts, by the InVITE Publications Committee (PC) that includes representatives from NIH and each of the country teams. The PC holds regular monthly meetings.

The study design and protocol were developed collaboratively with members from each participating country to ensure endpoints relevant within their country/region were included. The country teams are responsible for submitting for ethical revieiw, continuing review reports, quality management plans, protocol deviations, and serious adverse event reports per country regulations and as requested by NIH. Material Transfer Agreements for the transfer of InVITE specimens from the study sites to the central laboratory have been developed or are under development to transfer samples to the NIH for centralized laboratory testing. Half of the aliquots remain in-country. Central testing was determined to be optimal to limit interlaboratory variability and to have ability to use assays that may not be available in all locations such as neutralization assays with live viruses and full genome sequencing of SARS-CoV-2 variants.

The InVITE NIH protocol leads worked with the DCR Clinical Trials Research Section (CTRS) data management team to develop the case report forms (CRFs) and core standard operating procedure documents and subsequently with country teams to develop site-specific documents. To improve data quality and consistency, the CTRS data management team has been updating the original instructions for completion of CRFs if needed based on the changing environment and vaccine requirements in countries throughout the course of the study. As the CRF versions were finalized, the CTRS data management team built and validated the database. Any updates to the CRFs were also validated in the database before moving the database into production. Specimen kits with standard barcodes were designed for specimen collection. Detailed standard operating procedures for specimen kit preparation and use have been provided.

The individual sites collect the data on source documents or paper CRFs, prepare and review the CRFs for completion and submit them to the CTRS data management team via the database software. The CTRS data management team is responsible for all data entry across all sites. The CRFs are entered, tracked and any missing data or discrepancies are queried on an ongoing basis. The observational nature of this study allows use of a Quality Management Plan or a Monitoring Plan depending on the country requirements. Each plan was reviewed by the SP and protocol leads prior to the start of the study to be sure sites were adhering to the protocol, laboratory, and Good Clinical Practice requirements.

Study-related documents and CRFs, core standard operating procedures, and daily updated reports of study enrollment, visit status and general demographics can be viewed on the study’s restricted-access SharePoint site by staff from all sites. Individual country folders contain site-specific documents in their respective languages, as needed. The CTRS data management team provided training on the SharePoint site as each new site joined, and the team continues to provide training as needed.

The InVITE protocol team developed training materials including the general protocol overview, inclusion/exclusion criteria, study schedule, review of sample collection requirements and sample collection kits, data management and CRF completion instructions, review of Good Clinical Practice requirements and any site-specific appendix instructions required to conduct the study. The trainings were conducted virtually with the PI of each site/country and the staff designated to be involved in the study in attendance. The sites were required to fill out training attendance logs and return these to the InVITE study team. Refresher trainings are offered as needed.

**Fig 2:**
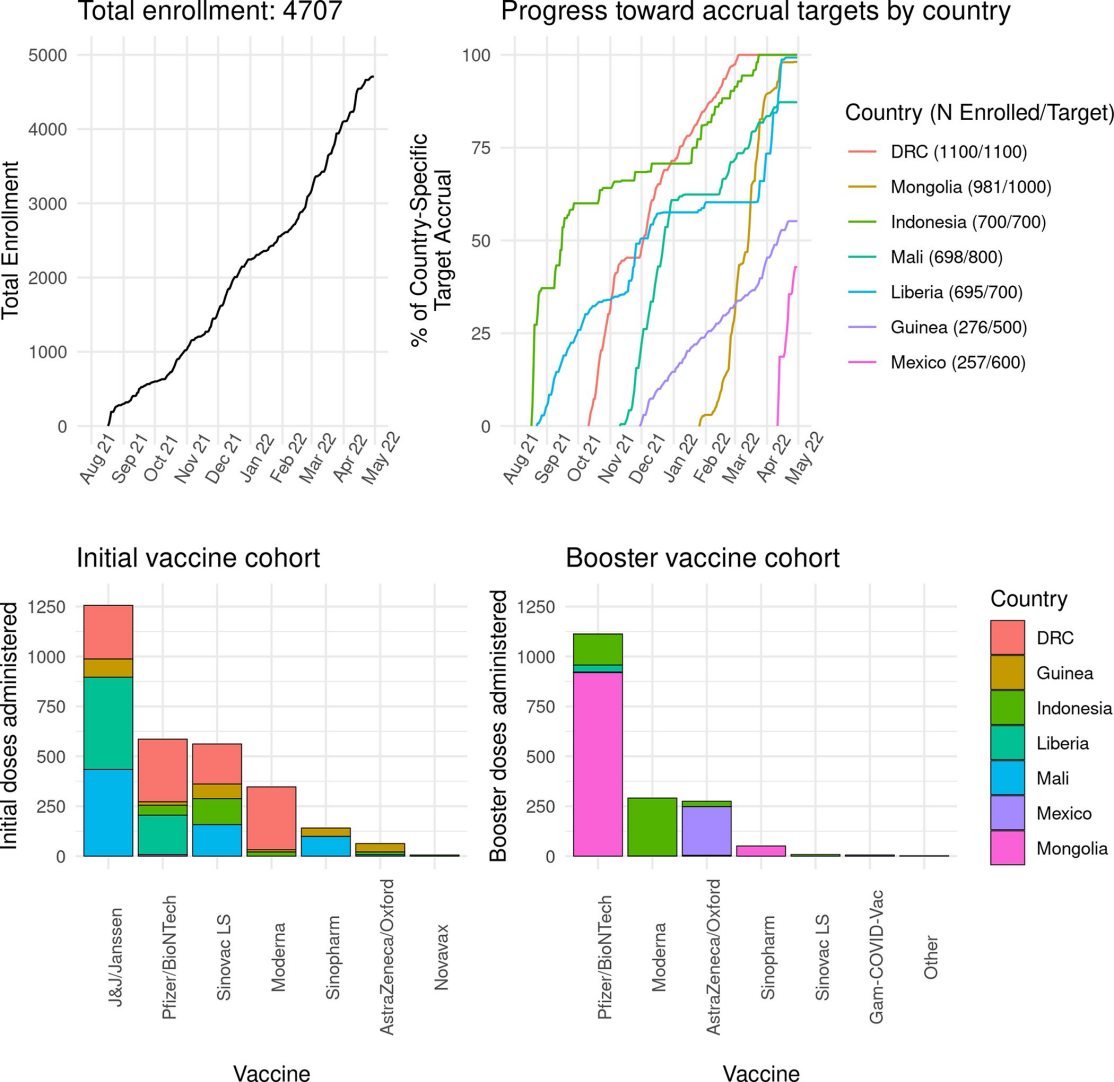
A. Current Enrollment Overall and by Country as of April 30, 2022. Analysis population includes participants with complete data on receipt of initial or booster vaccine regimen, Visit 1 date, and manufacturer of first vaccine who met all eligibility criteria, provided informed consent within one day of receiving a COVID-19 vaccine, and were deemed eligible to continue to Visit 2. Data cleaning is ongoing as the study continues. **B. Vaccines Received to Date in the Initial and Booster Cohorts as of April 30, 2022.** Analysis population includes participants with complete data on receipt of initial or booster vaccine regimen, Visit 1 date, and manufacturer of first vaccine who also met all eligibility criteria, provided informed consent within one day of receiving a COVID-19 vaccine, and were deemed eligible to continue to Visit 2. Data cleaning is ongoing as the study continues.

The study was launched on August 16, 2021, and the first participant enrolled on August 18, 2021. The study is currently enrolling. As of April 30, 2022, 4707 participants have been enrolled (Fig 2A) and the distribution by country and vaccine type is shown in Fig 2B. A total of 2961 people have been enrolled as primary vaccine series and a total of 1746 as boosters (either 3^rd^ or even 4^th^ dose).

## Discussion

We have designed and launched an international multi-country observational cohort study of COVID-19 vaccine immunogenicity across several vaccine platforms. We will be able to evaluate immunogenicity of different vaccines, durability of immune responses, and the impact of demographics and comorbidities on humoral immune responses as well as detailed assessment of COVID-19 infections occurring during study participation. This international effort will provide immunogenicity data for multiple vaccines used in the study countries under the conditions of program implementation. Despite logistical challenges that included submissions to multiple ethics committees, cross-continent conference calls, coordination of sample collection, storage, and shipping worldwide, supply chain shortages and CRF development, the study is successfully enrolling, and we anticipate reporting preliminary results within this calendar year. We believe that this type of study can help provide important data including within and between country comparisons of vaccine platforms that may help inform public health decisions, planning and implementation. In addition, it could facilitate collection of useful data on determinants of immune responses, susceptibility to breakthrough infections and potential immune correlates, durability of antibody titers and differences in immunogenicity seen among different vaccine regimens.
