# Correction: Design of an observational multi-country cohort study to assess immunogenicity of multiple vaccine platforms (InVITE)

**DOI:** 10.1371/journal.pone.0314048

**Published:** 2024-11-14

**Authors:** Irini Sereti, Kathryn Shaw-Saliba, Lori E. Dodd, Robin L. Dewar, Sylvain Laverdure, Shawn Brown, Olivier Tshiani Mbaya, Jean Jacques Muyembe Tamfum, Placide Mblala-Kingebeni, Ydrissa Sow, Esther Akpa, Mory Cherif Haidara, Karine Fouth Tchos, Abdoul Habib Beavogui, Aaron Neal, Dona Arlinda, Dewi Lokida, Louis Grue, Mary Smolskis, Laura A. McNay, Dehkontee Gayedyu-Dennis, Guillermo M. Ruiz-Palacios, Abelardo Montenegro-Liendo, Moctar Tounkara, Seydou Samake, Ganbolor Jargalsaikhan, Delgersaikhan Zulkhuu, Shera Weyers, Tyler Bonnett, Gail E. Potter, Randy Stevens, Adam Rupert, Jamila Aboulhab, Jean-Luc Biampata, Alexandre Delamou, Bienvenu Salim Camara, Herman Kosasih Indonesia, Muhammad Karyana, James T. Duworko, Justino Regalado-Pineda, Paola del Carmen Guerra-de-Blas, Seydou Doumbia, Djeneba Dabitao, Naranjargal Dashdorj, Naranbaatar Dashdorj, Kevin Newell, Alyson Francis, Kevin Rubenstein, Victoria Bera, Iman Gulati, Ratna Sardana, Monica Millard, Renee Ridzon, Sally Hunsberger

The 35^th^ author’s name is spelled incorrectly. The correct name is: Alexandre Delamou.
